# Addressing global health priorities: Balancing investments in existing and emerging approaches

**DOI:** 10.7189/jogh.01.010101

**Published:** 2012-06

**Authors:** Igor Rudan, Ana Marušić, Harry Campbell

## Abstract

One of the common themes in contemporary global health is finding an optimal balance between investments in existing and emerging approaches to fight global health priorities. Existing interventions have been proven to be effective, but they usually have limitations. Emerging interventions could potentially bring greater gains at a lower cost, but health gains are usually uncertain and take much more time to achieve. There are no simple solutions on how to balance funding support to these two competing approaches, but some components of successful strategies are becoming increasingly apparent. Transparency over the expected return on investment, style of investment and time horizon can assist rational investment decisions.

One of the common themes in contemporary global health is finding an optimal balance between investments in existing and emerging approaches to fight global health priorities [1]. Existing interventions that have been proven to be effective can be scaled up at a certain cost to provide additional health gains, but they usually have limitations. Supporting the development of novel (emerging) interventions could potentially bring greater gains at a lower cost, but health gains are usually uncertain and take much more time to achieve. There are no simple solutions on how to balance funding support to these two competing approaches in order to achieve greatest gains at the lowest cost within a defined period of time [2]. However, some components of successful strategies are beginning to seem increasingly apparent. As a starting point, we could pose this question: why should anyone choose to invest in either scaling up existing health interventions, or developing new ones? Any investment can typically be linked to an expectation of the investor for some return on the investment. What can be seen as the return on investment in this case? This probably depends on who the investors are. Governments and international agencies are expected to use taxpayer’s money to reduce the overall disease burden in a cost-effective way. Industry, however, may be primarily interested in generating patents on discoveries that could secure financial profit from future sales of both existing and emerging interventions. Not-for-profit organizations and private donors may have their own specific priorities that do not necessarily need to be either rational or transparent [3,4]. When balancing investments in existing and emerging health interventions, investors need to carefully consider the style of investing they wish to adopt. Among an incredibly broad set of options, investors can choose to support only one or a subset of them; and can adopt a predominantly risk-neutral, risk-averting or risk-seeking approach. Governments are typically expected to adopt a risk-neutral approach and diversify their support across a set of proven existing interventions, while also identifying a few promising emerging approaches which they would like to introduce in the future. Industry would be more likely to adopt a risk-averting strategy by minimizing support to complex downstream research and focusing on improvements to existing interventions, while carefully selecting the most promising emerging ones that are already in the pipeline for investment. Private donors may adopt a risk-seeking strategy by focusing on a very specific target within a set time frame. They may be in a position to invite the most original ideas and out-of-the-box thinking that could revolutionize global health and eradicate the problem entirely, while accepting the risk that most of the funding will ultimately fail to result in any progress at all [5]. The time-frame within which investors expect a return on their investment is another critically important factor to consider. When the investment context is one of perceived urgency or of a short time horizon for action to achieve returns on investment, the balance will be heavily skewed toward support for implementing and upgrading existing interventions. If the investment context is one with a much longer-term horizon then the balance will shift toward more uncertain, higher risk options, which hold the promise of considerably greater benefits per unit of cost [6,7]. In this issue, we present several papers that closely relate to these issues. An expert opinion exercise conducted by Bahl et al. focused on setting research priorities to reduce the global burden of preterm birth and low birth weight [8]. Rudan et al. present research priorities among emerging interventions against major childhood infections, as determined by a multidisciplinary panel of international experts [9]. Chopra et al. describe and discuss the complex interplay between the determinants of cost-effectiveness and equity when planning the scale-up of health interventions that can achieve child mortality reduction [10]. Finally, Feng et al. assemble a unique and large data set on a broad range of health and socio-economic variables and then use multivariable approaches in an attempt to understand the relative contributions of a range of recent health and social changes within Chinese society to the dramatic reduction of child mortality which has occurred during the period 1990-2006 [11].
